# Reply to ‘Increases in temperature do not translate to increased flooding’

**DOI:** 10.1038/s41467-019-13613-4

**Published:** 2019-12-12

**Authors:** Jiabo Yin, Pierre Gentine, Shenglian Guo, Sha Zhou, Sylvia C. Sullivan, Yao Zhang, Lei Gu, Pan Liu

**Affiliations:** 1 State Key Laboratory of Water Resources and Hydropower Engineering Science, Wuhan University, Wuhan, 430072 China; 2 Department of Earth and Environmental Engineering, Columbia University, New York, NY 10027 USA; 3 Earth Institute, Columbia University, New York, NY 10025 USA; 4 Lamont-Doherty Earth Observatory of Columbia University, Palisades, New York, NY 10964 USA

**Keywords:** Climate change, Hydrology, Hydrology, Natural hazards

**Replying to** Wasko et al. *Nature Communications* https://doi.org/10.1038/s41467-019-13613-4 (2019)

We are thankful for the interest of Wasko et al.^1^ in our work^2^. The correspondence from Wasko et al. argued that our finding on the positive scaling rate between storm runoff extremes and temperature can be mainly attributed to snowmelt processes, and claimed that storm runoff extremes should have a negative scaling rate globally. However, we do not agree with their arguments for several reasons.

First, Wasko et al. stated that our use of daily storm runoff extremes is not a perfect indicator of flash flooding characteristics. We agree with Wasko et al. that minute or hour-flow discharge data are better able to capture the details of the flooding generation process. Unfortunately, a global higher-resolution streamflow observation network is not available and a daily dataset can still capture the essence of fast flow extremes. The flooding generations governed by both extreme storms and underlying surface conditions are complex interacting phenomena, which are inherently characterized by peak discharge, flood volume and hydrograph shape, particularly in natural hazard risk assessment and water resources management. The daily streamflow extremes, having a high correlation with peak discharge, represent a key readily available measure of extreme storms and are frequently used to assess flood hazard risk^3^. Many recent studies have reported that sub-daily extremes are usually more sensitive to temperatures than those at a daily scale^4^, so the strong positive scaling rates of storm runoff extremes detected in our original publication^2^ may probably further increase at a finer temporal resolution. In addition, inspired by the comment of Wasko et al., we used the high-quality continental US MOPEX dataset and a Chinese basin dataset for detecting and matching the precipitation and streamflow peaks in the same storm events, identifying these two peaks occurring within 7 days, and then conduct scaling analysis with the coincident temperature at the catchment scales. With the matched precipitation and streamflow peaks, we could still observe strong positive scaling rates of storm runoff extremes, and these results are robust when changing the extreme definition or using daily mean temperature (Tmean) 1 day prior to the precipitation peak (Supplementary Figs. 1–4).

Second, Wasko et al. defended our regression fitting only up to the peak point temperature (*T*_pp_) and associated different *T*_pp_ between precipitation and storm runoff extremes. Wasko et al. misunderstood and assumed that we presupposed that both streamflow and precipitation must increase together with rising temperature. On the contrary, we did not make such an assumption. Instead we quantified and estimated the *T*_pp_ for both precipitation and storm runoff extremes, independently. With this method, we did not guarantee that the *T*_pp_ of precipitation and storm runoff were the same. Wasko et al. show an example in their Fig. 1b, c of the different temperature ranges of precipitation and storm runoff extremes. In reality, the large difference they plot is not representative of our study, as our observations in Fig. 2 of our primary publication^2^ show that the differences of *T*_pp_ are usually within 1–2 °C in most areas of the globe except for limited regions in western United States and Southern Europe. Why do we need to detect the *T*_pp_ for precipitation and storm runoff extremes individually? Extreme precipitations are mainly governed by atmospheric thermodynamics and partially modulated by atmospheric dynamics, while the runoff generation is also impacted by underlying surface conditions. As the *T*_pp_ varies widely from region to region, spanning 0–30 °C^5^, we detected the *T*_pp_ station by station using the LOWESS method for precipitation and storm runoff extremes, and then used this to partition the non-monotonic hook structure into two branches (ascending below and descending above). We also examined the scaling pattern with the high-quality continental US MOPEX dataset and a Chinese basin dataset, and further reconfirmed the positive scaling rates and *T*_pp_ variability at the smaller catchment scale (Supplementary Figs. 5–8).

**Fig. 1:**
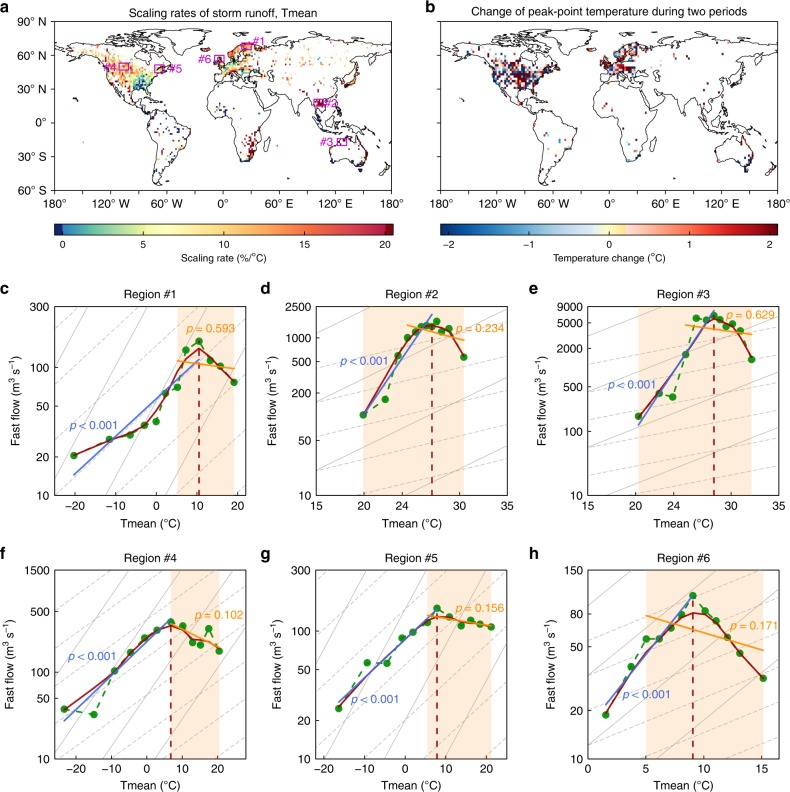
Scaling rates and change in peak point temperature of 99th percentile storm runoff extremes with local temperature. **a** Scaling rate results published in ref. ^2^. **b** Change of peak point temperature from 1961–1990 to 1991–2017. **c**–**h** Scaling curve of example station in different regions. Green scatters in **c**–**h** are 99th percentile extremes in temperature bins, and red curves are the fitted hook structures using a LOWESS method; vertical red dashed line indicates the peak point temperature, and blue (or orange) lines and *p*-value is obtained by our method (or method in ref. ^15^). The shading shows the temperature range used in ref. ^15^. The Clausius–Clapeyron (C–C) scaling is shown in light grey dashed lines, and 2CC in light grey solid lines.

**Fig. 2:**
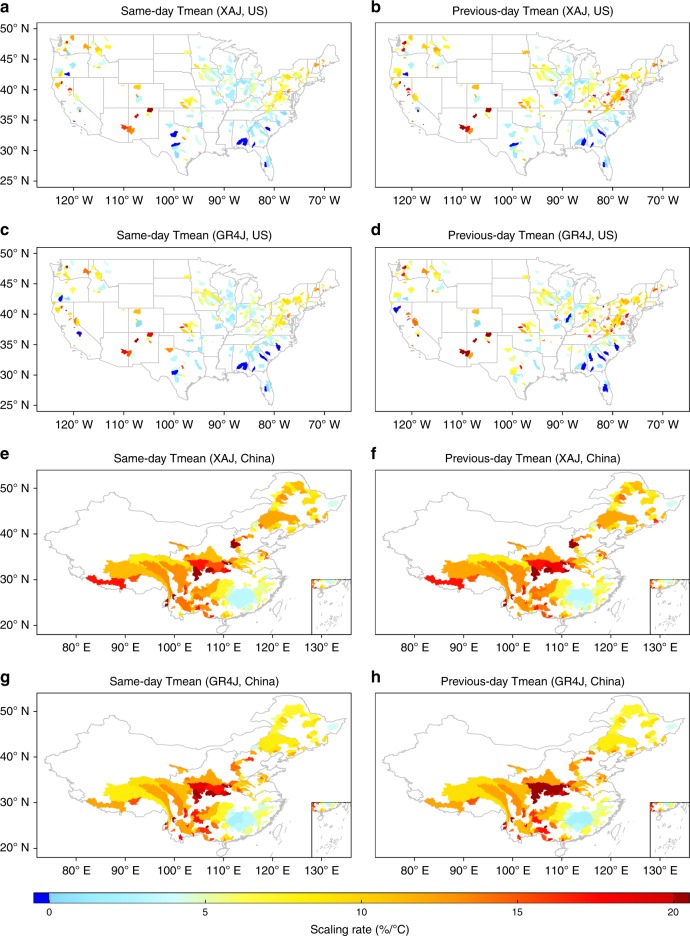
Scaling rates of simulated runoff extremes over the US and China. Scaling rates of 99th percentile simulated rain-induced runoff extremes by hydrological models with same-day (or previous-day) temperatures at the catchment scale over US and China. **a**, **b** Scaling rates for XAJ model in the US; **c**, **d** Scaling rates for GR4J model in the US; **e**, **f** Scaling rates for XAJ model over China; **g**, **h** Scaling rates for GR4J model over China.

To further emphasize the importance of considering hook structures, we focus on six regions in Fig. 1c–h to show the importance of considering such hook structures. Our linear fitting is statistically significant at a 0.001 level, while neglecting such hook structures seldomly passes the significance test (Supplementary Fig. 9). We note that our publication^2^ and previous study^6^ have explicitly explained that the hook structure does not imply a potential upper limit for future extremes, as the change of peak point temperature will shift the hook curve toward warmer temperatures in the future (Fig. 1b). We refer the interested readers to those publications for details on the underlying physical mechanisms.

Third, Wasko et al. argued that snow runoff would explain our observed temperature relationship. We believe this is incorrect. Snow runoff mostly contributes to base flow expect for snowmelt-dominated regions, which were explicitly removed from our analysis, whereas our analysis is focusing on the fast flow component. In fact, we explicitly took off base flow from our analysis as it is known to be affected by land-use land cover changes, land management, water usage and also by the modification of plant transpiration by increased [CO_2_]^7^. In the scaling analysis over cold regions in Fig. 1, we observe that the phase change to ice (snow) does not change the scaling pattern of storm runoff response. Additionally, the latent heat of melting is almost an order of magnitude less than the latent heat of vaporization^8^, so that the phase change of snow would have limited impacts on the scaling rates of snow fall compared to rainfall. Even if snowmelt might play an important role in runoff generation, this can only be applied in snowmelt-dominated regions, which does not account for a major fraction of the northern hemisphere (Supplementary Fig. 10). Moreover, global warming may induce a shift toward low snow years, thus resulting in high early-season snowmelt and runoff, implying increasing flood risk due to snowmelt-dominated role in runoff generation over snow-dependent regions^9^.

We attempted to examine the impact of snowmelt on storm runoff extremes using a multifaced approach. First, we tested the impact of the snowmelt temperature and omitted the data colder than 1.0 °C, 2.0 °C and 5.0 °C, respectively, and we also extracted data warmer than 0 °C for removing possible snowpack runoff consideration. In all cases, we still observed widespread positive scaling rates between storm runoff extremes and temperatures under different scenarios (Supplementary Figs. 11–14 and Supplementary Table 1), further confirming our earlier findings. Second, we detected the occurrence month of storm runoff extremes for each year, and identified the snowmelt season for each year with the first month period (or 2-month period, 3-month period) during which the mean temperature exceeding 0 °C. After deriving the probability of annual storm runoff extremes occurring in snowmelt season with the long-term series, we find that the peak events have low occurrence likelihood to be impacted by snowmelt over the majority of the globe (Supplementary Fig. 15). In the few regions where the peak events occur in the winter (Supplementary Fig. 16), snow is rare (e.g., Southeast US, Spain, Western France) and not the main cause of flooding, which is rather due to synoptic weather patterns^10^. Our conclusion still holds true when we define the snowmelt season with the 1.0 °C melting temperature, further proving that snowmelt did not have a large impact on extremes and let alone the positive scaling rates. Third, we selected two hydrological models to segment the rain-induced runoff and snowmelt runoff. One is the GR4J hydrological model, which contains a CemaNeige snowmelt module, and the other is the Xinanjiang (XAJ) hydrological model incorporated with the snowfall–snowmelt module in Soil and Water Assessment Tool (SWAT); these have been widely employed in capturing snowfall and snowmelt processes in hydrological community. The high Kling-Gupta efficiency (KGE) value and the similar scaling results of simulated storm runoff extremes with those of observations both verified the good simulation performance of these two models (Supplementary Figs. 17 and 18). After excluding the snowmelt component of runoff by hydrological models, the simulated rain-induced runoff extremes also yielded a strong positive scaling rate despite the fact that it is slightly smaller than that of storm runoff extremes for a few catchments (Fig. 2 and Supplementary Table 2). Last, we examined the change in the scaling curve over six example basins spanning both cold and warm regions, confirming that the hook structure would shift toward a warmer side and thus resulting in an intensification of hydrological extremes in a warmer world (Supplementary Fig. 19). This also provides strong evidence that the snowmelt impacts did not challenge our earlier finding of positive scaling rates between storm runoff extremes and temperatures.

Finally we would like to conclude by pointing out that, although the scaling-based projection is based on historical changes projected forward, a key conclusion in our publication that the storm runoff extremes may increase under warming in the future is not challenged by Wasko et al. and is supported by prevailing projections about increasing flood risk in the climate community^11^. The inconsistency between our publication and Wasko et al. is not due to snowmelt consideration but due to their omission of the consideration of a hook temperature structure and separation of base flow. Wasko et al. claimed that a decrease in antecedent soil moisture and snowmelt under a warming climate may somewhat offset flood intensification. However, precipitation intensity is the dominant driver of flood hazards^12^, and soil moisture is not projected to change dramatically^13^, except in transitional regions. We also find a significant seasonal variability of soil moisture; although both surface and root-zone soil moistures show a slight decreasing change in winter, they are more likely increasing in summer and autumn over most regions of the globe (Supplementary Figs. 20 and 21). In addition, the storm runoff extremes rarely occurred in winter (Supplementary Fig. 16), and in the regions where it occurs in the winter have very rare snow occurrences (e.g., Southeastern US, Spain, Western France). Instead, the nonlinear increase in runoff coefficient^14^ and land-use land cover changes such as forest degradation contribute likely more to fast flow extreme intensification. We would like to thank Wasko et al. for this correspondence, which helped clarify several of our findings and we hope that it will generate more investigation of flash flooding generation mechanisms.

## Supplementary information

Supplementary Information

## Data Availability

The primary data are available from ref. ^2^. The MOPEX data are available from National Oceanic and Atmospheric Administration website (https://www.nws.noaa.gov/ohd/mopex/mo_datasets.htm). The weekly snow cover data are from Northern Hemisphere EASE-Grid 2.0 Weekly Snow Cover and Sea Ice Extent, Version 4, which is archived in National Snow & Ice Data Center (https://nsidc.org/). The global gridded Berkeley Earth Surface Temperatures (BEST) dataset is from Berkeley Earth (http://berkeleyearth.org/). The soil moisture data are from the Global Land Evaporation Amsterdam Model (GLEAM) version 3 (https://www.gleam.eu). The high-resolution (0.5° × 0.5°) gridded daily precipitation and temperature dataset in China is obtained from Chinese Meteorological Administration (http://www.cma.gov.cn/). The streamflow data of Chinese river basins are available from the authors upon request.
