# Supplementary material for: Reply to ‘Increases in temperature do not translate to increased flooding’
Source: Nat Commun. 2019 Dec 12;10:5675. doi: 10.1038/s41467-019-13613-4 (PMC6908579; doi:10.1038/s41467-019-13613-4)
Supplement: Supplementary file 1 — Supplementary Information [file 41467_2019_13613_MOESM1_ESM.pdf]

SUPPLEMENTARY INFORMATION

Reply to ‘Increases in temperature do not translate to increased flooding’

Yin et al.

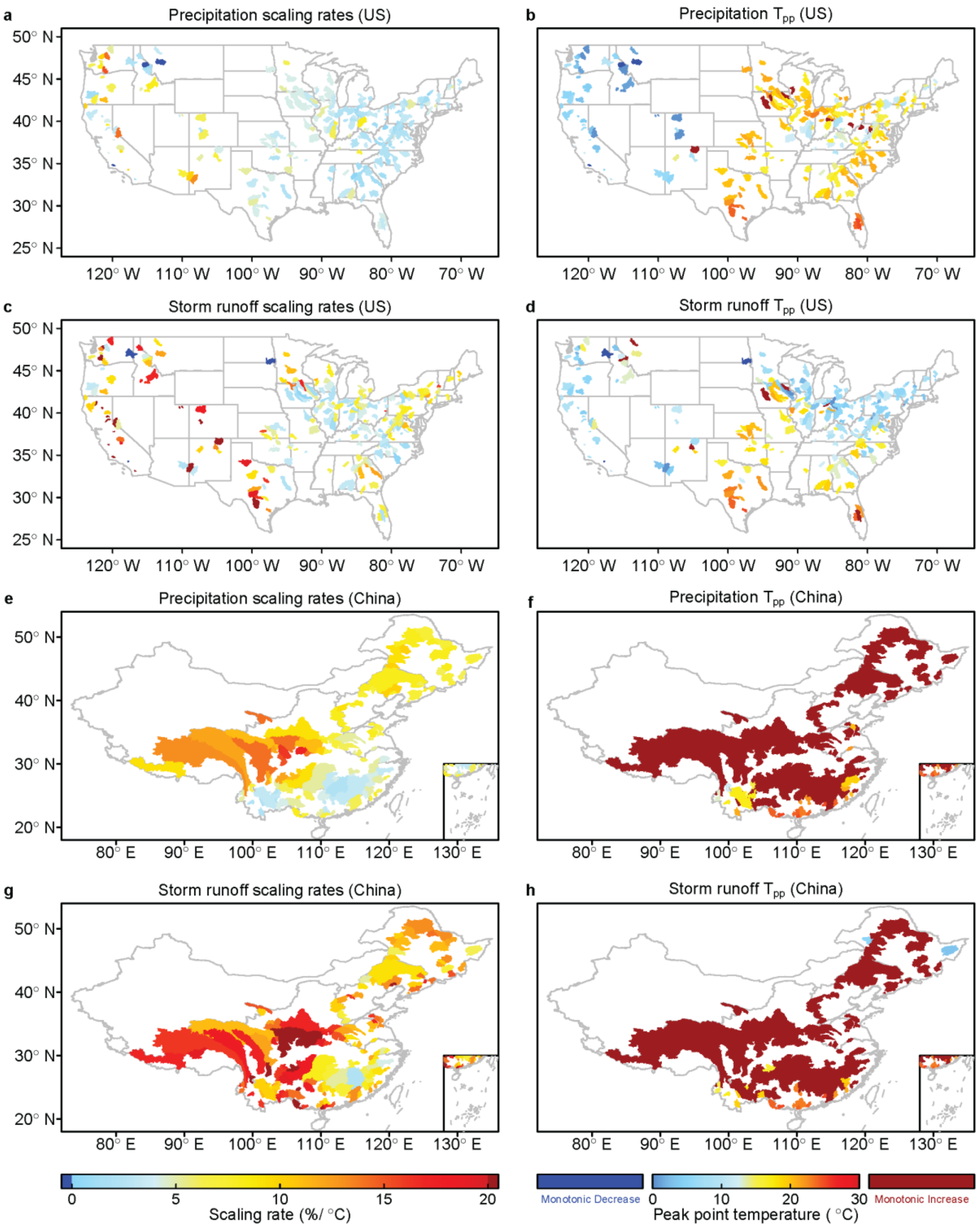

Supplementary Figure 1 | Scaling results and peak point temperature of 99th percentile precipitation and storm runoff extremes with same-day temperature at the catchment scale over US and China. a-d, scaling rates and peak point temperatures of MOPEX dataset in US. **e-h**, scaling rates and peak point temperatures over China. The precipitation and storm runoff peaks within same events are extracted and matched, and then are used in scaling analysis.

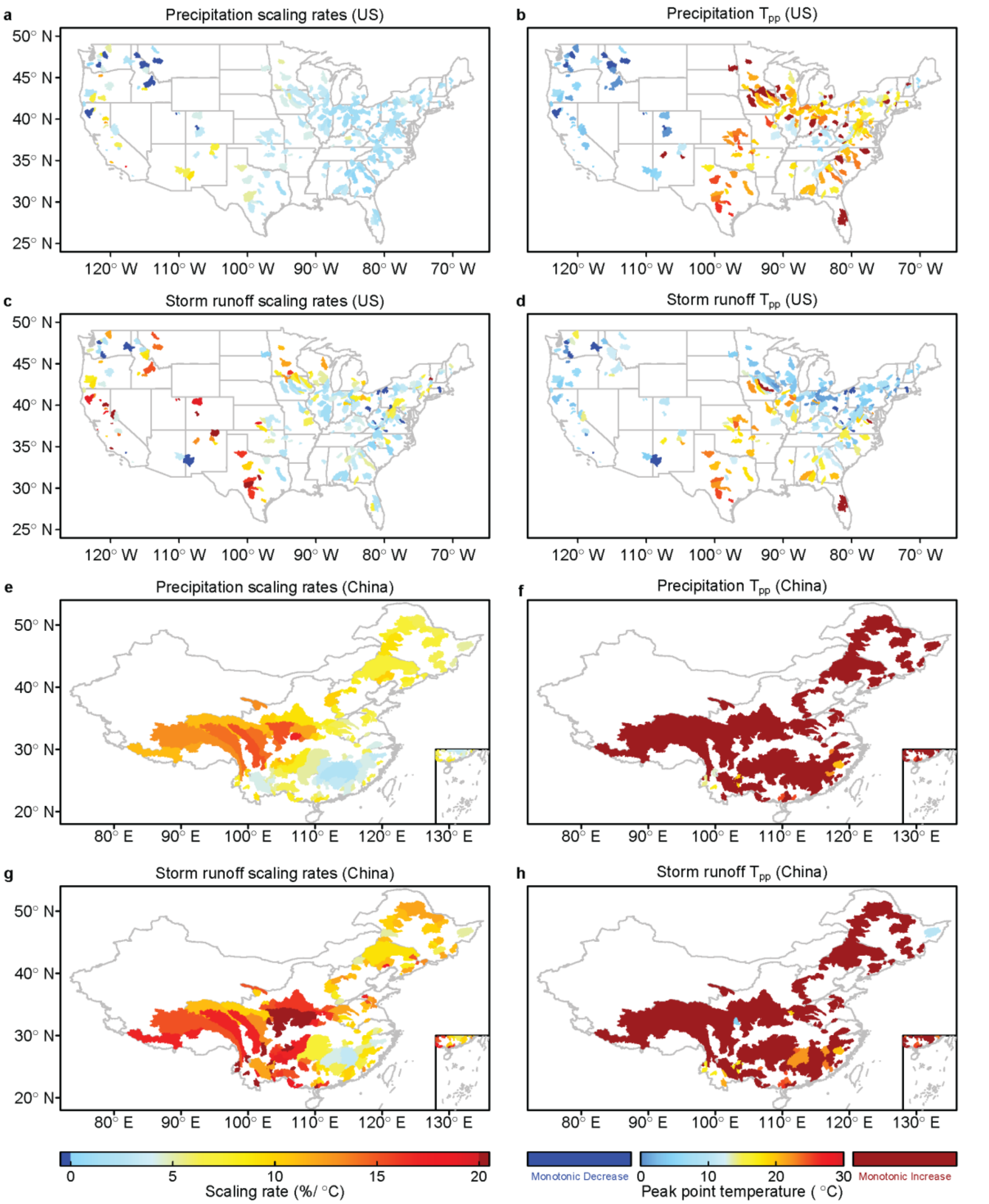

Supplementary Figure 2 | Scaling results and peak point temperature of 99th percentile precipitation and storm runoff extremes with previous-day temperature at the catchment scale over US and China. a-d, scaling rates and peak point temperatures of MOPEX dataset in US. **e-h**, scaling rates and peak point temperatures over China. The precipitation and storm runoff peaks within same events are extracted and matched, and then are used in scaling analysis.

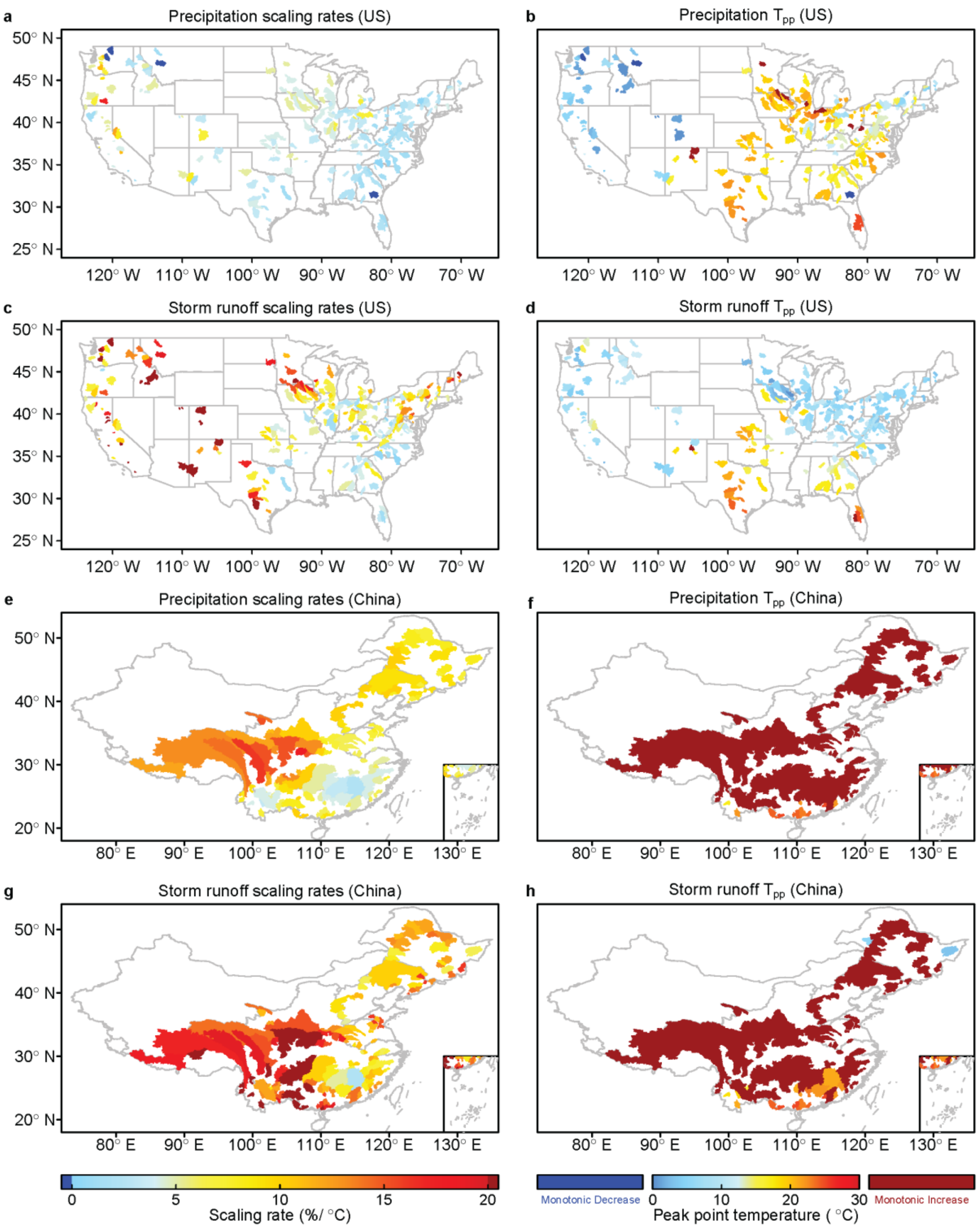

Supplementary Figure 3 | Scaling results and peak point temperature of 95th percentile precipitation and storm runoff extremes with same-day temperature at the catchment scale over US and China. a-d, scaling rates and peak point temperatures of MOPEX dataset in US. **e-h**, scaling rates and peak point temperatures over China. The precipitation and storm runoff peaks within same events are extracted and matched, and then are used in scaling analysis.

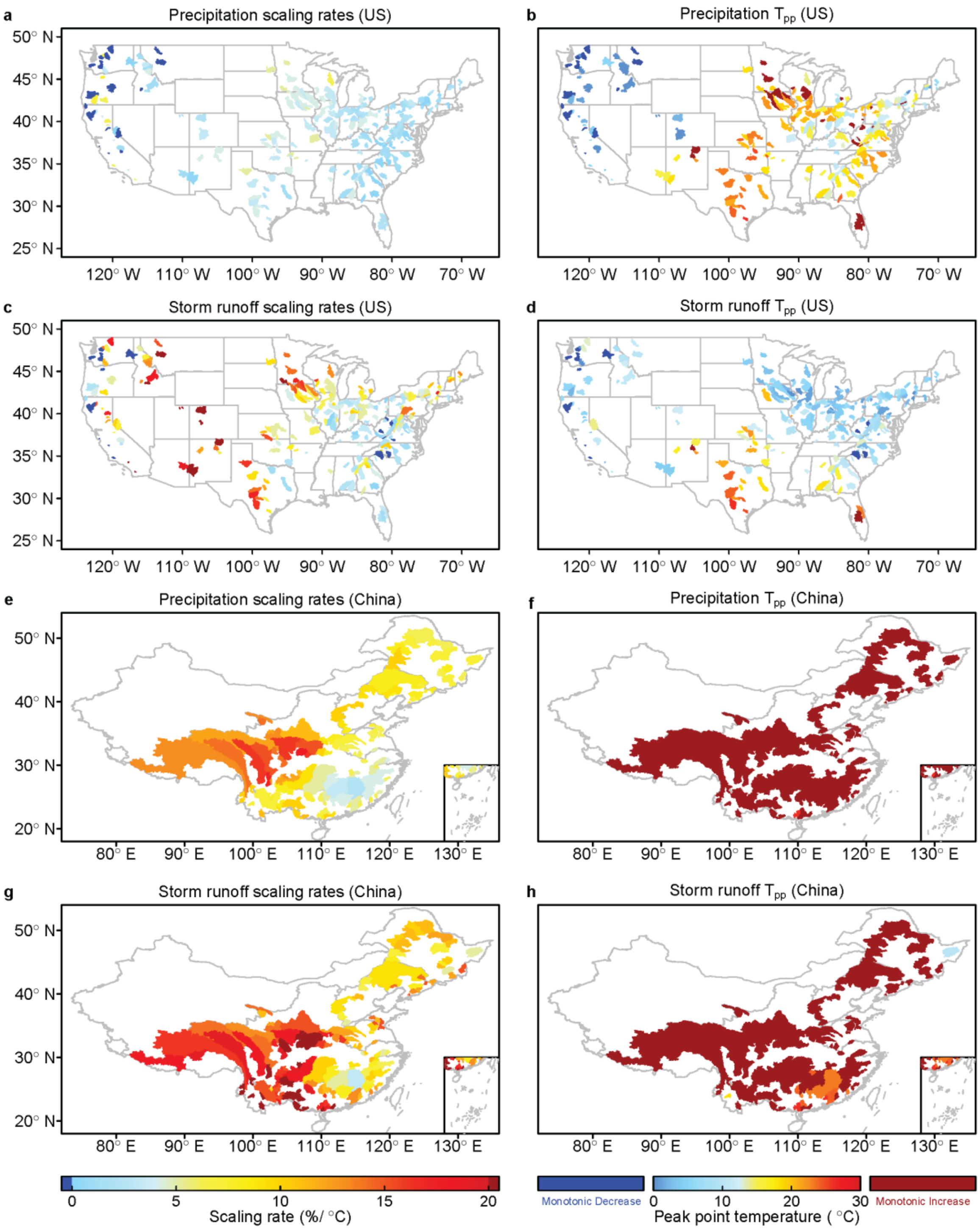

Supplementary Figure 4 | Scaling results and peak point temperature of 95th percentile precipitation and storm runoff extremes with previous-day temperature at the catchment scale over US and China. a-d, scaling rates and peak point temperatures of MOPEX dataset in US. **e-h**, scaling rates and peak point temperatures over China. The precipitation and storm runoff peaks within same events are extracted and matched, and then are used in scaling analysis.

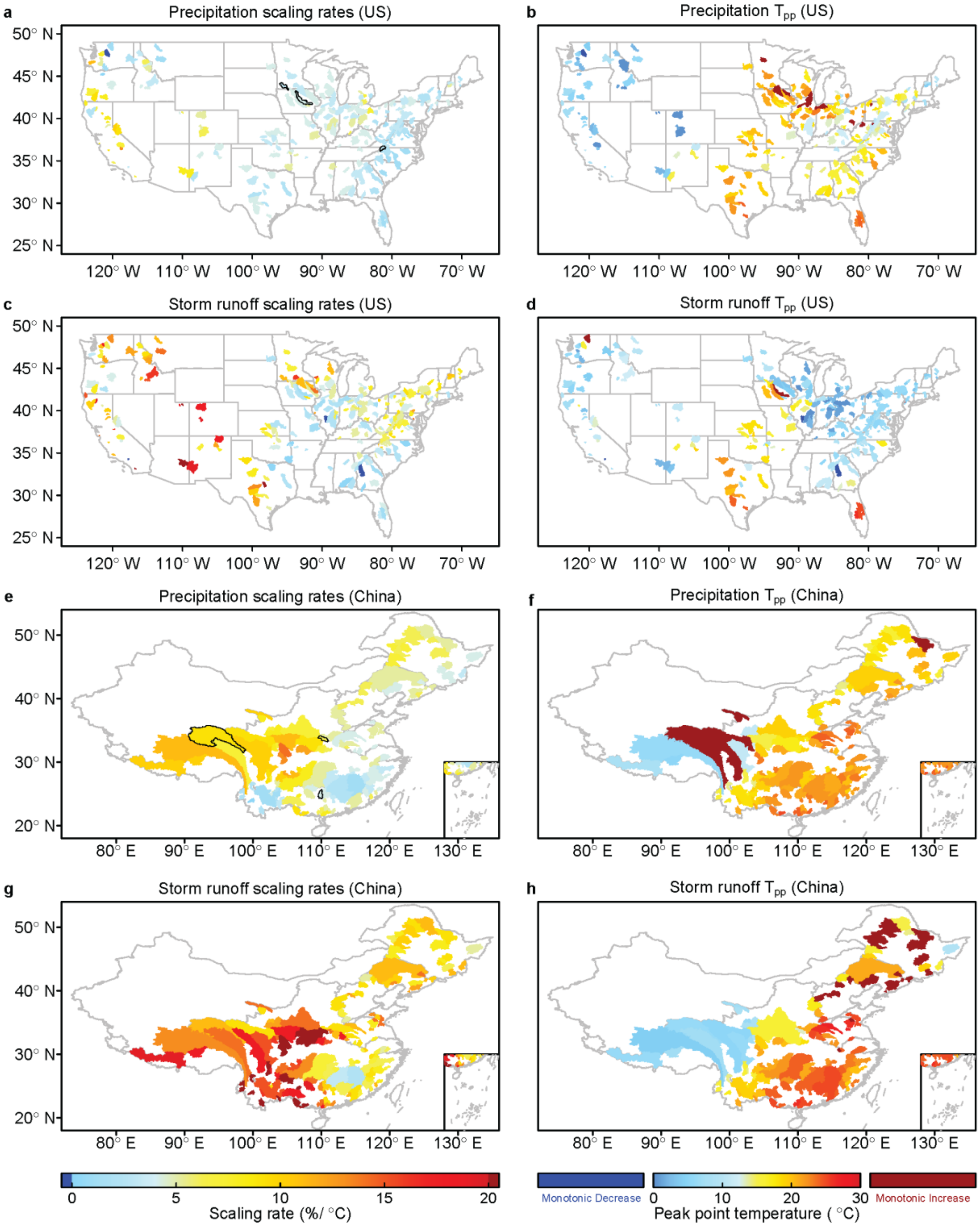

Supplementary Figure 5 | Scaling results and peak point temperature of 99th percentile precipitation and storm runoff extremes with same-day temperature at the catchment scale over US and China. a-d, scaling rates and peak point temperatures of MOPEX dataset in US. **e-h**, scaling rates and peak point temperatures over China.

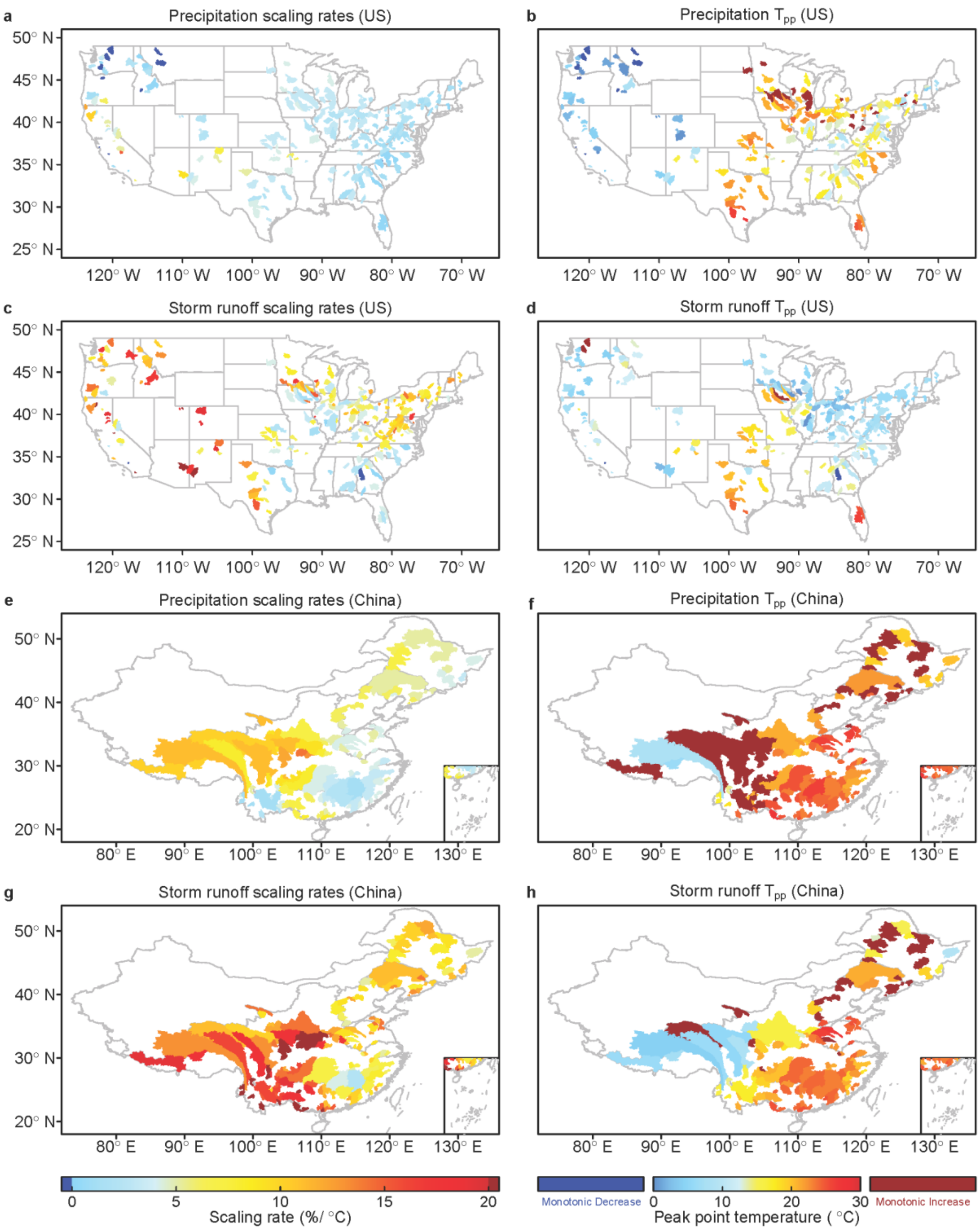

Supplementary Figure 6 | Scaling results and peak point temperature of 99th percentile precipitation and storm runoff extremes with pervious-day temperature at the catchment scale over US and China. a-d, scaling rates and peak point temperatures of MOPEX dataset in US. **e-h**, scaling rates and peak point temperatures over China.

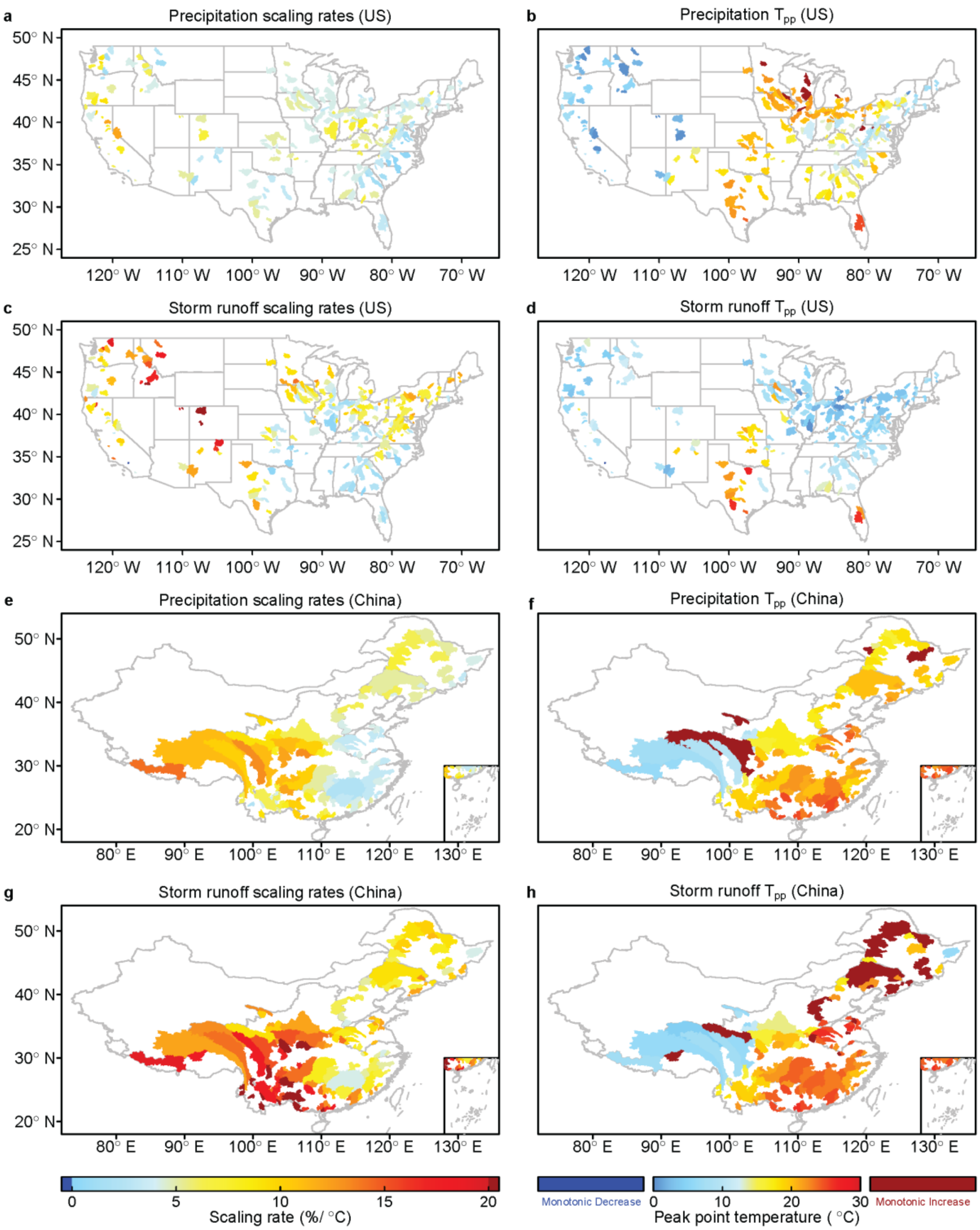

Supplementary Figure 7 | Scaling results and peak point temperature of 95th percentile precipitation and storm runoff extremes with same-day temperature at the catchment scale over US and China. a-d, scaling rates and peak point temperatures of MOPEX dataset in US. **e-h**, scaling rates and peak point temperatures over China.

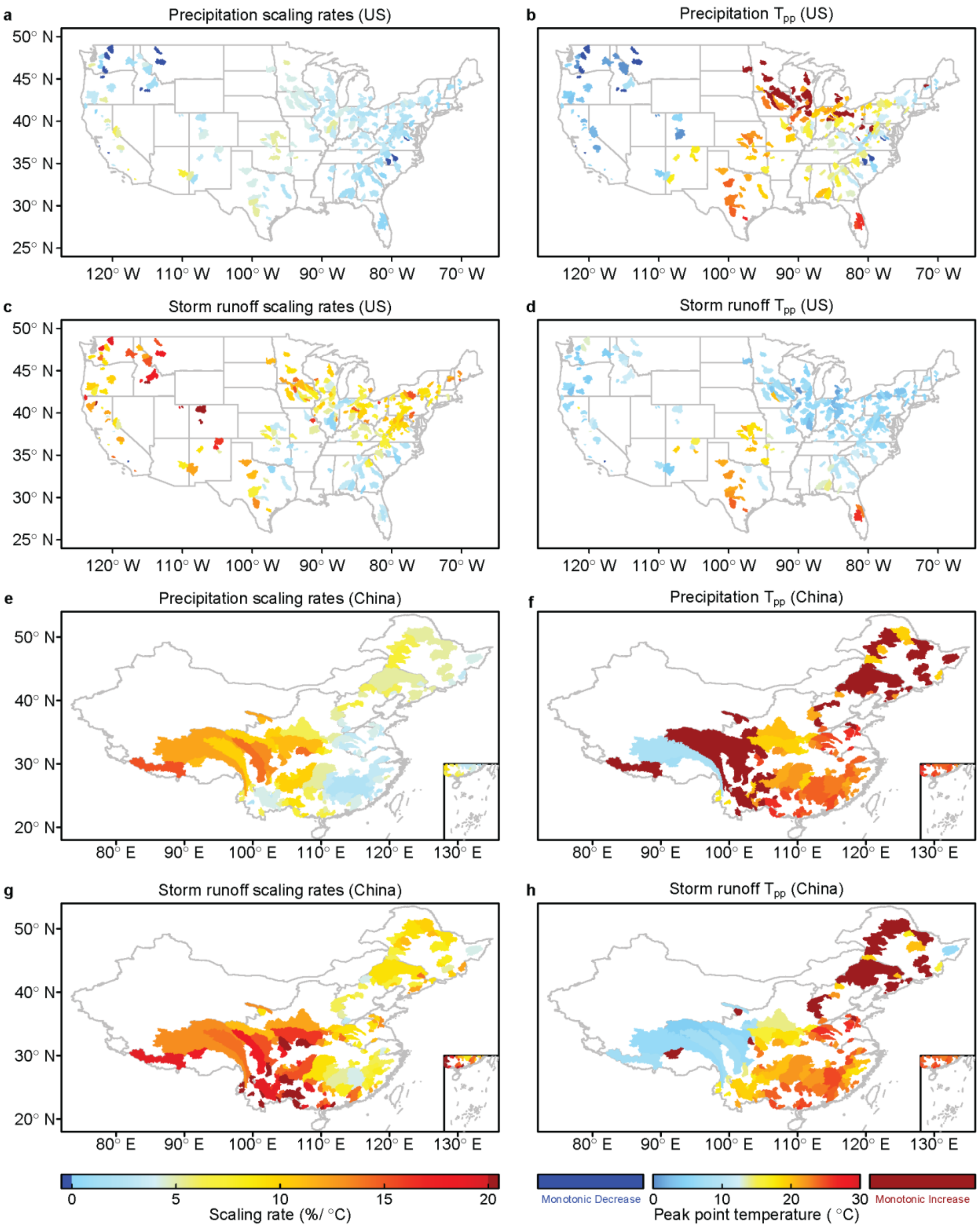

Supplementary Figure 8 | Scaling results and peak point temperature of 95th percentile precipitation and storm runoff extremes with previous-day temperature at the catchment scale over US and China. a-d, scaling rates and peak point temperatures of MOPEX dataset in US. **e-h**, scaling rates and peak point temperatures over China.

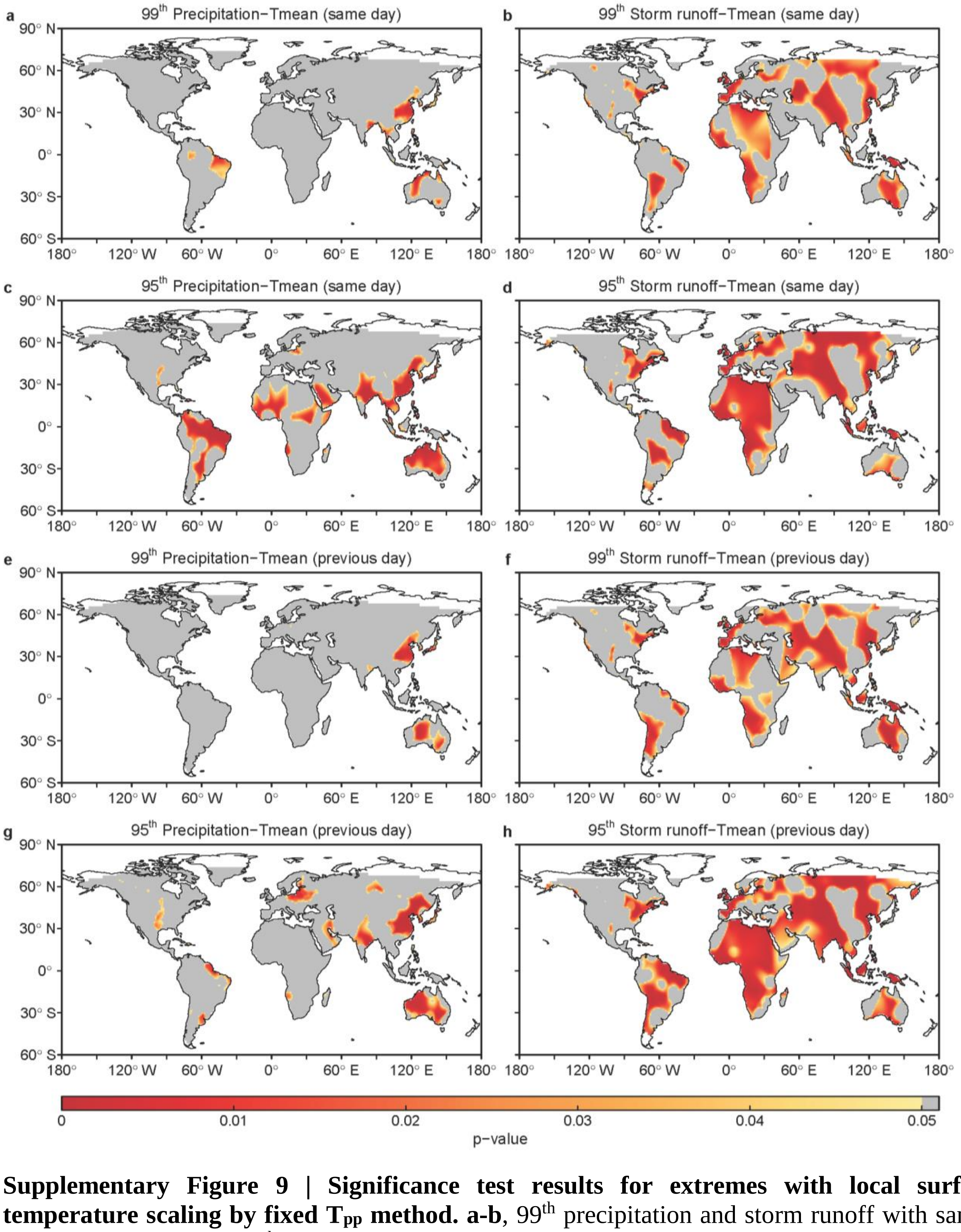

Supplementary Figure 9 | Significance test results for extremes with local surface temperature scaling by fixed T_{pp} method. a-b, 99th precipitation and storm runoff with same-day temperature. **c-d**, 95th precipitation and storm runoff with same-day temperature. **e-f**, 99th precipitation and storm runoff with previous-day temperature. **g-h**, 95th precipitation and storm runoff with previous-day temperature. This figure is interpolated using a bilinear method.

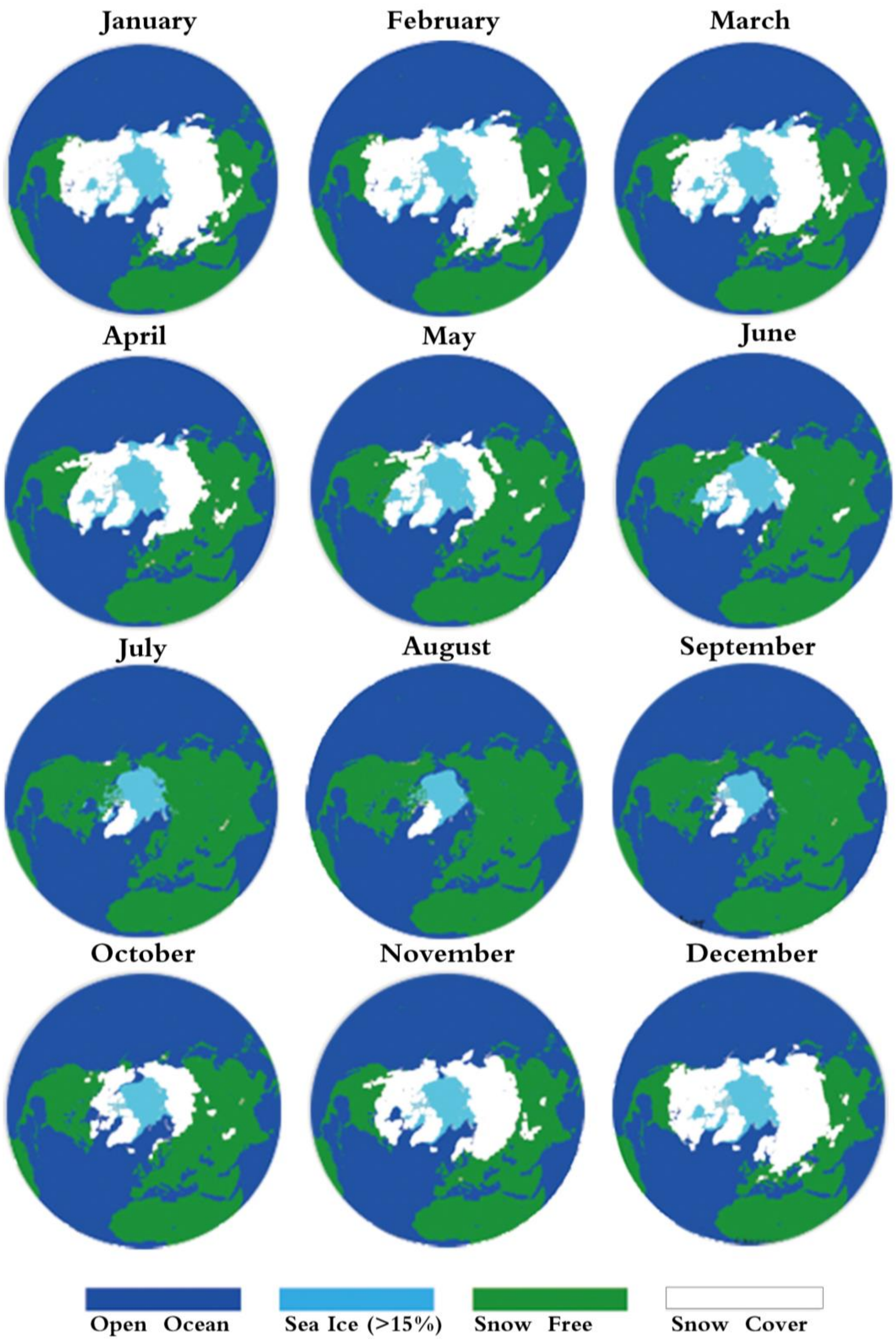

Supplementary Figure 10 | Map of snow cover regions during 1966-2018. This map is cited and adjusted from the dataset of Northern Hemisphere EASE-Grid 2.0 Weekly Snow Cover and Sea Ice Extent, Version 4. The weekly snow cover data is archived in National Snow & Ice Data Center (NSIDC).

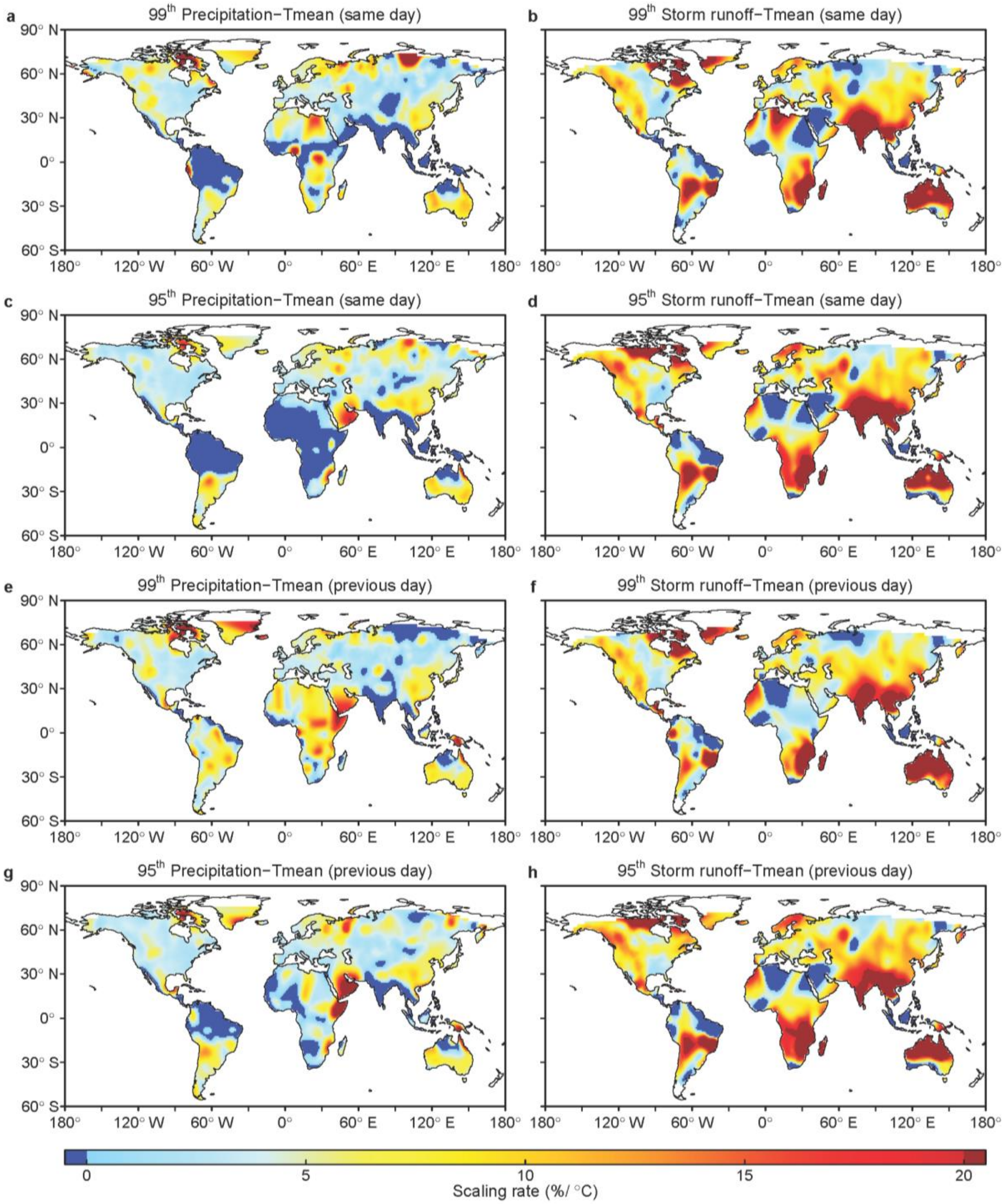

Supplementary Figure 11 | Scaling rates of extreme with daily mean temperature when excluding data with temperature colder than 0 °C. a-b, 99th precipitation and storm runoff with same-day temperature. **c-d**, 95th precipitation and storm runoff with same-day temperature. **e-f**, 99th precipitation and storm runoff with previous-day temperature. **g-h**, 95th precipitation and storm runoff with previous-day temperature. This figure is interpolated using a bilinear method.

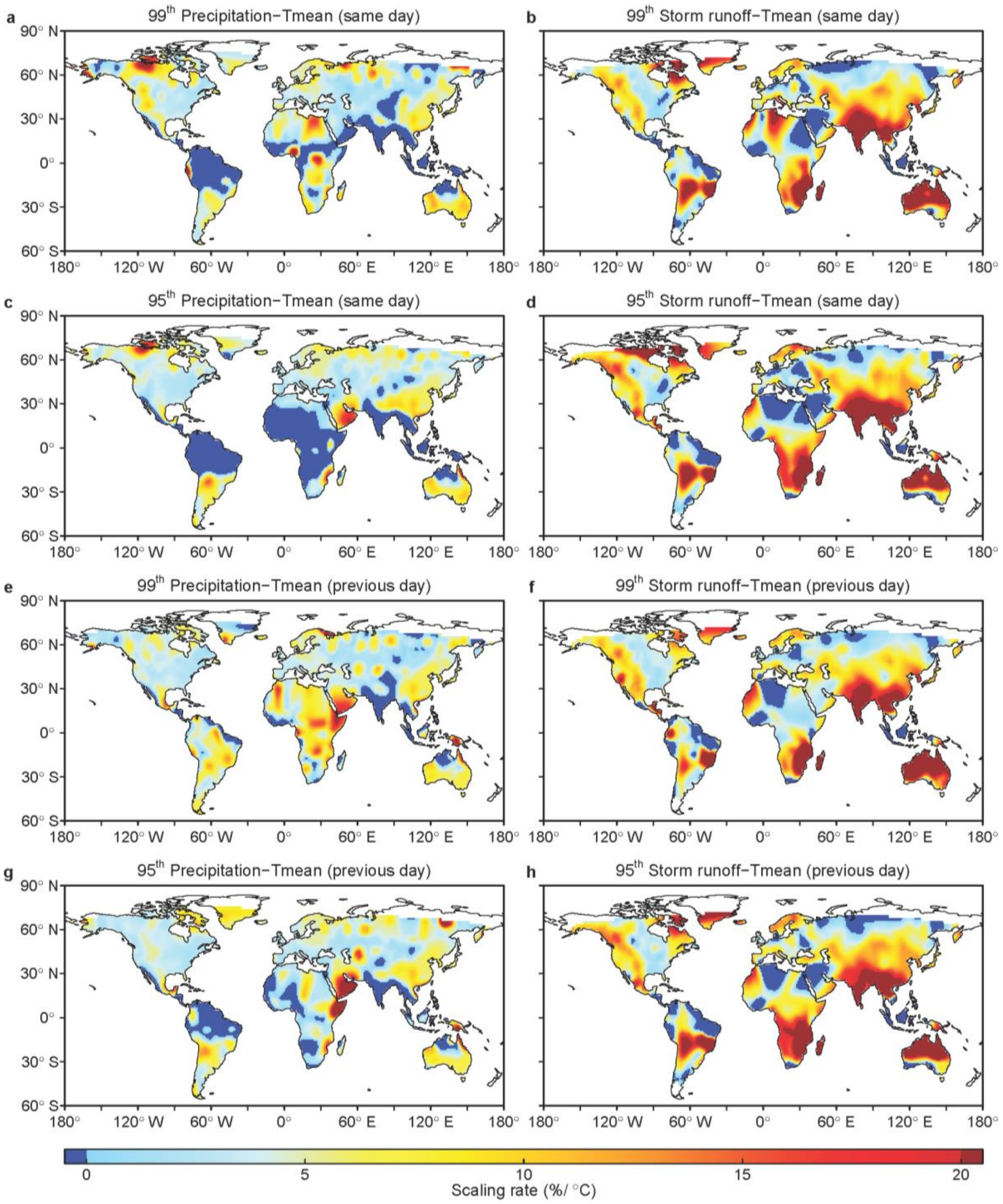

Supplementary Figure 12 | Scaling rates of extreme with daily mean temperature when excluding data with temperature colder than 1 °C. a-b, 99th precipitation and storm runoff with same-day temperature. **c-d**, 95th precipitation and storm runoff with same-day temperature. **e-f**, 99th precipitation and storm runoff with previous-day temperature. **g-h**, 95th precipitation and storm runoff with previous-day temperature. This figure is interpolated using a bilinear method.

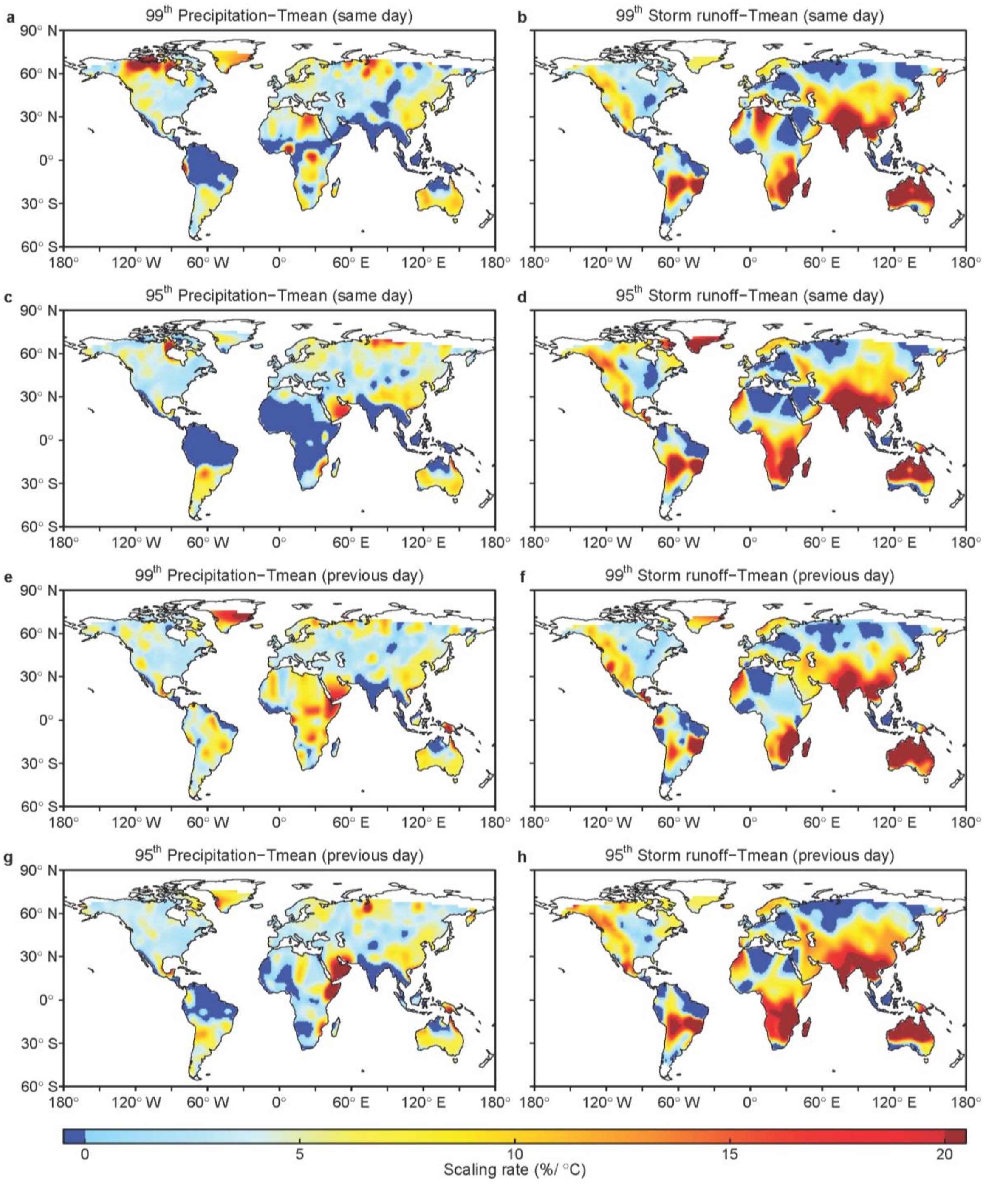

Supplementary Figure 13 | Scaling rates of extreme with daily mean temperature when excluding data with temperature colder than 2 °C. a-b, 99th precipitation and storm runoff with same-day temperature. **c-d**, 95th precipitation and storm runoff with same-day temperature. **e-f**, 99th precipitation and storm runoff with previous-day temperature. **g-h**, 95th precipitation and storm runoff with previous-day temperature. This figure is interpolated using a bilinear method.

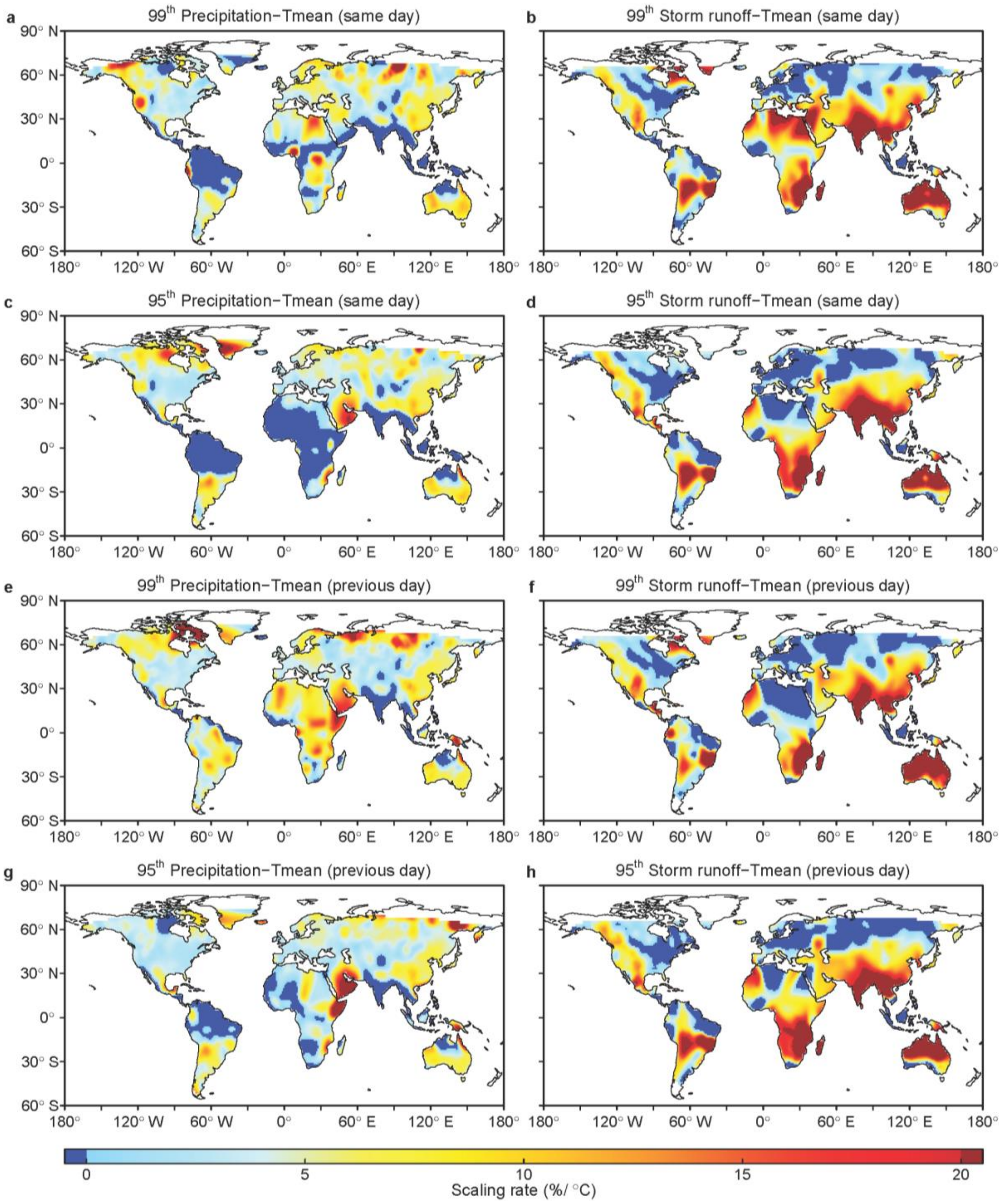

Supplementary Figure 14 | Scaling rates of extreme with daily mean temperature when excluding data with temperature colder than 5 °C. a-b, 99th precipitation and storm runoff with same-day temperature. **c-d**, 95th precipitation and storm runoff with same-day temperature. **e-f**, 99th precipitation and storm runoff with previous-day temperature. **g-h**, 95th precipitation and storm runoff with previous-day temperature. This figure is interpolated using a bilinear method.

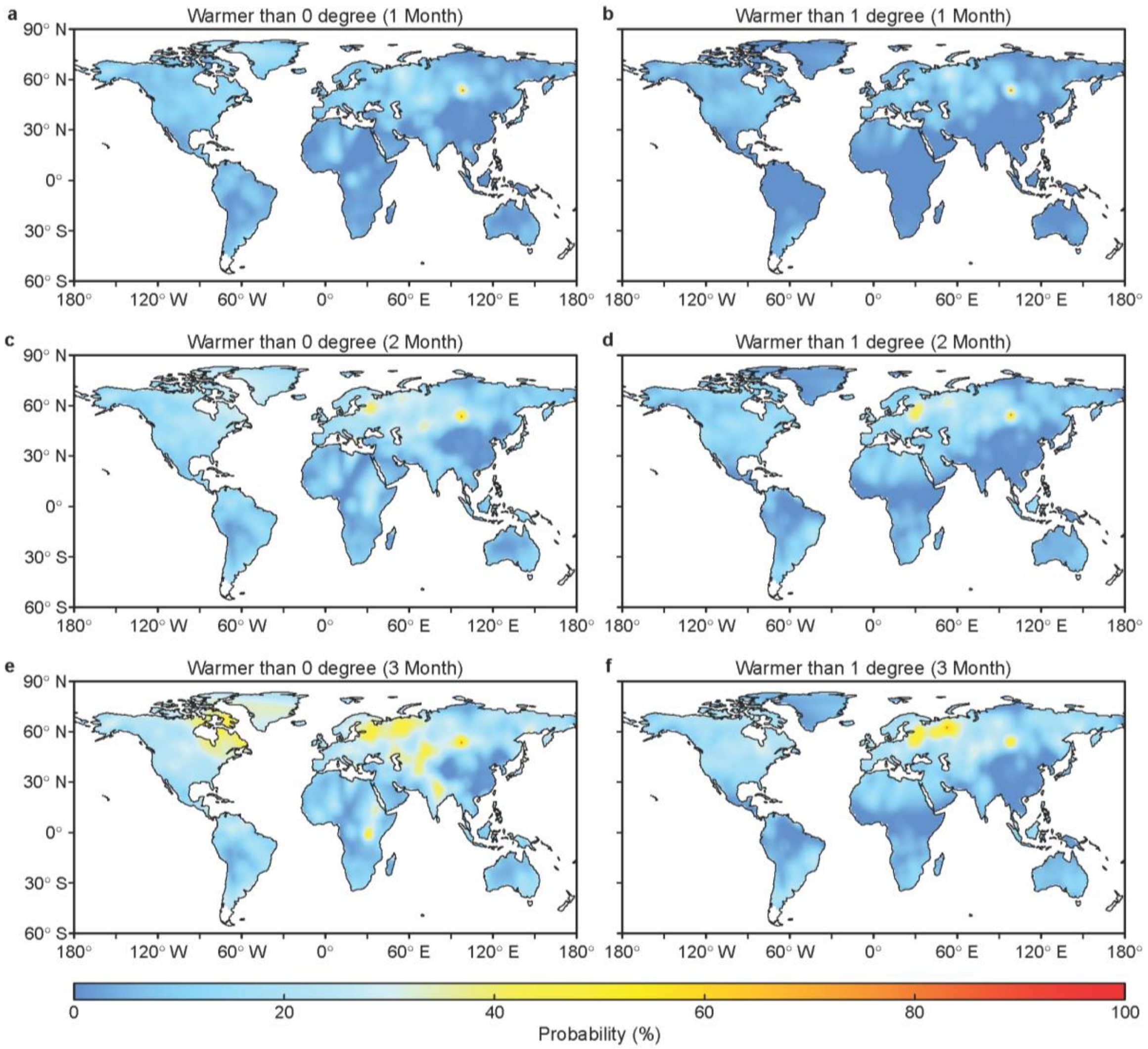

Supplementary Figure 15 | The probability of annual storm runoff extremes occurring in the snow melt season. **a-b**, the snow melt season is defined as the first month with temperature exceeding 0 °C (**a**), and 1 °C (**b**). **c-d**, the snow melt season is defined as the first two-month period with temperature exceeding 0 °C (**c**), and 1 °C (**d**). **e-f**, the snow melt season is defined as the first three-month period with temperature exceeding 0 °C (**e**), and 1 °C (**f**). The streamflow data is from GRDC and Chinese dataset, and the monthly gridded temperature data is from the Berkeley Earth Surface Temperatures (BEST) gridded dataset. The BEST data is at 1x1 degree, covering the period of 1850-2018. This figure is interpolated using a bilinear method.

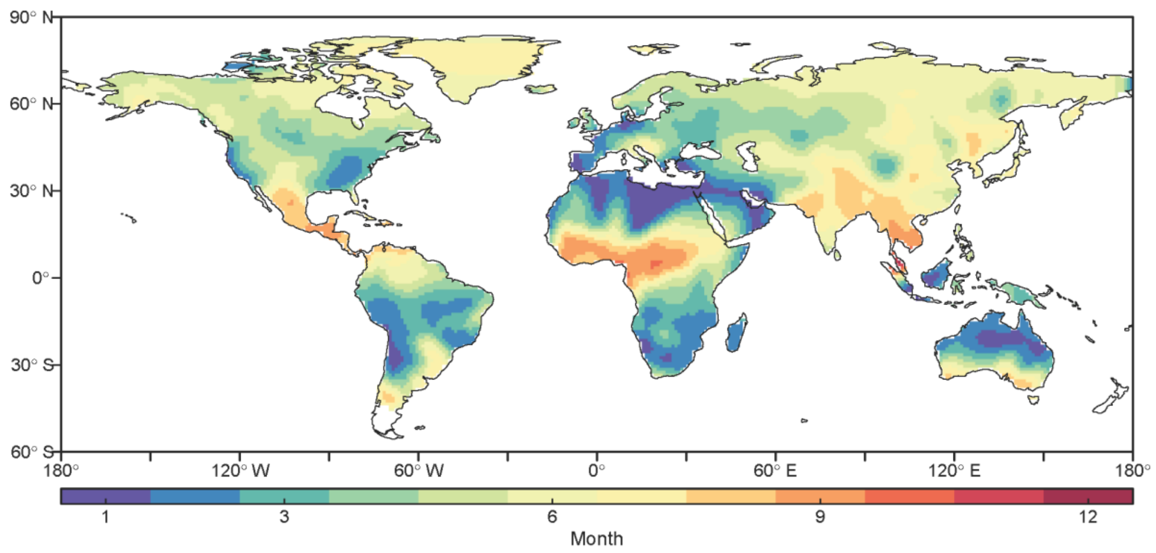

Supplementary Figure 16 | The average occurrence month of annual 99th percentile storm runoff extremes. The streamflow data is from GRDC and Chinese dataset. This figure is interpolated using a bilinear method.

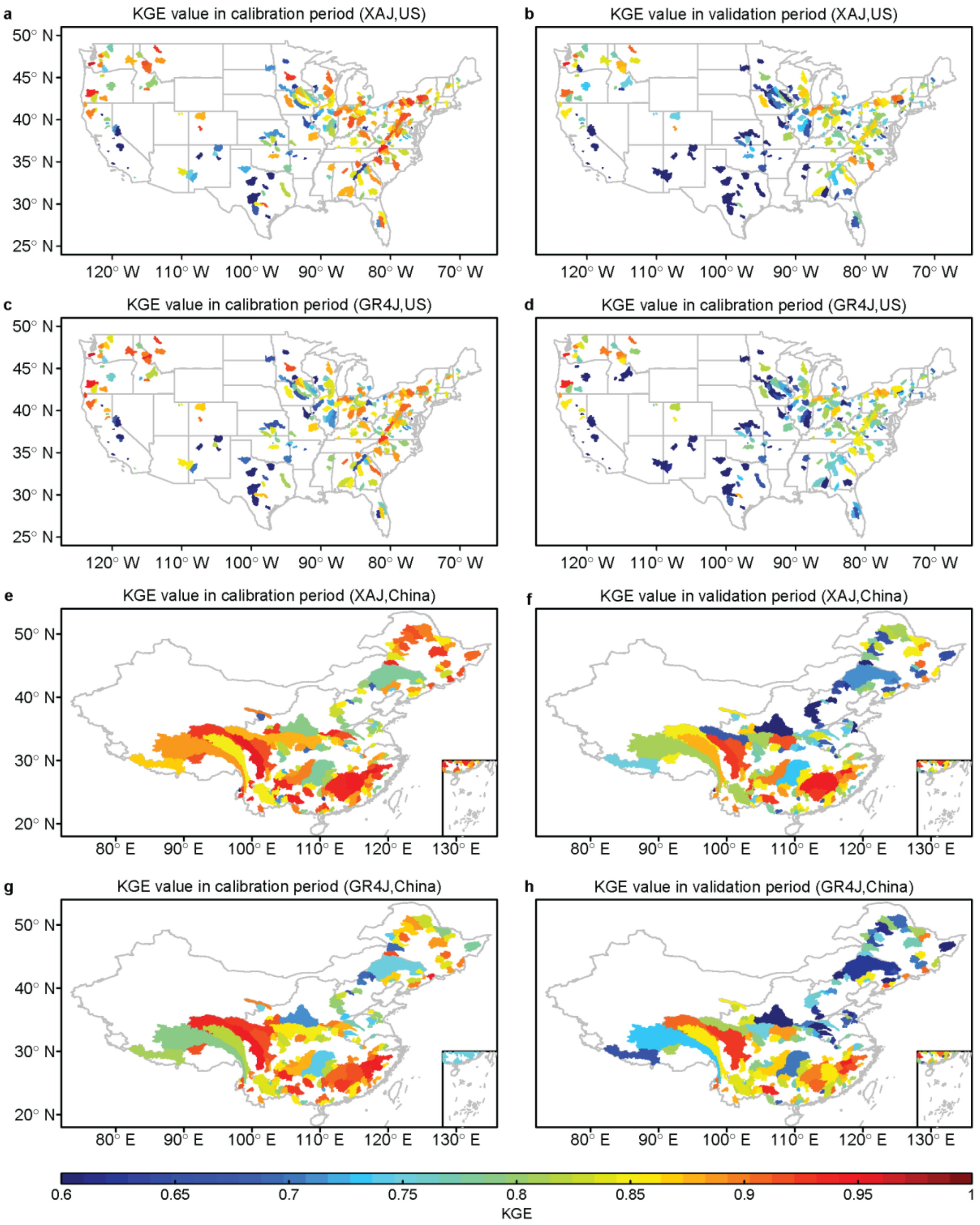

Supplementary Figure 17 | The KGE values during calibration and validation periods for US and China catchments. a-b, KGE value for XAJ model of MOPEX dataset in US. **c-d**, KGE value for GR4J model of MOPEX dataset in US. **e-f**, KGE value for XAJ model over China. **g-h**, KGE value for GR4J model over China. The 20-year observation period with highest data quality is used for model calibration and validation, and the calibration period accounts for 50%.

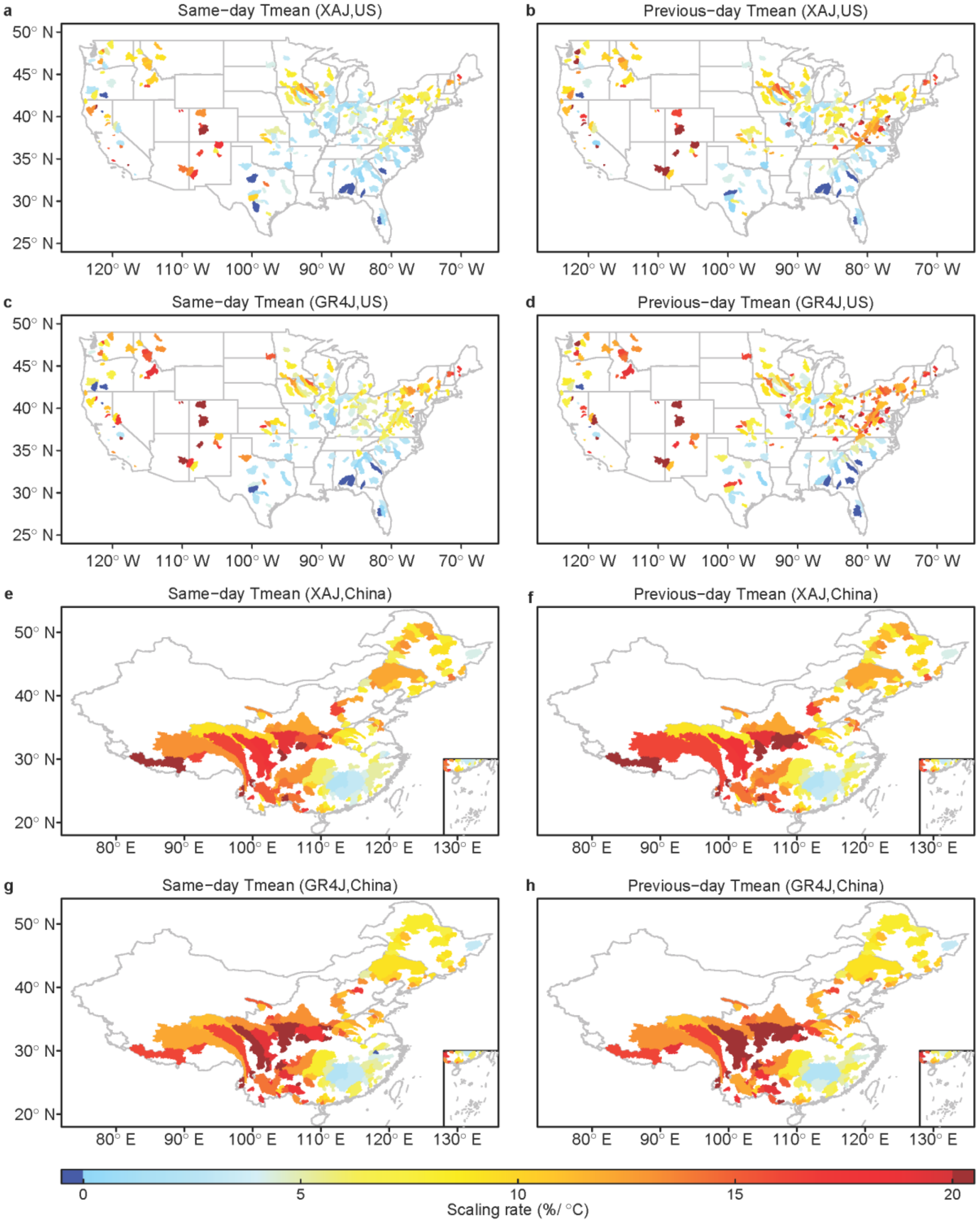

Supplementary Figure 18 | Scaling rates of 99th percentile simulated storm runoff extremes by hydrological models with same-day (or previous-day) temperatures at the catchment scale over US and China. a-b, scaling rates for XAJ model in the US; **c-d**, scaling rates for GR4J model in the US; **e-f**, scaling rates for XAJ model over China; **g-h**, scaling rates for GR4J model over China.

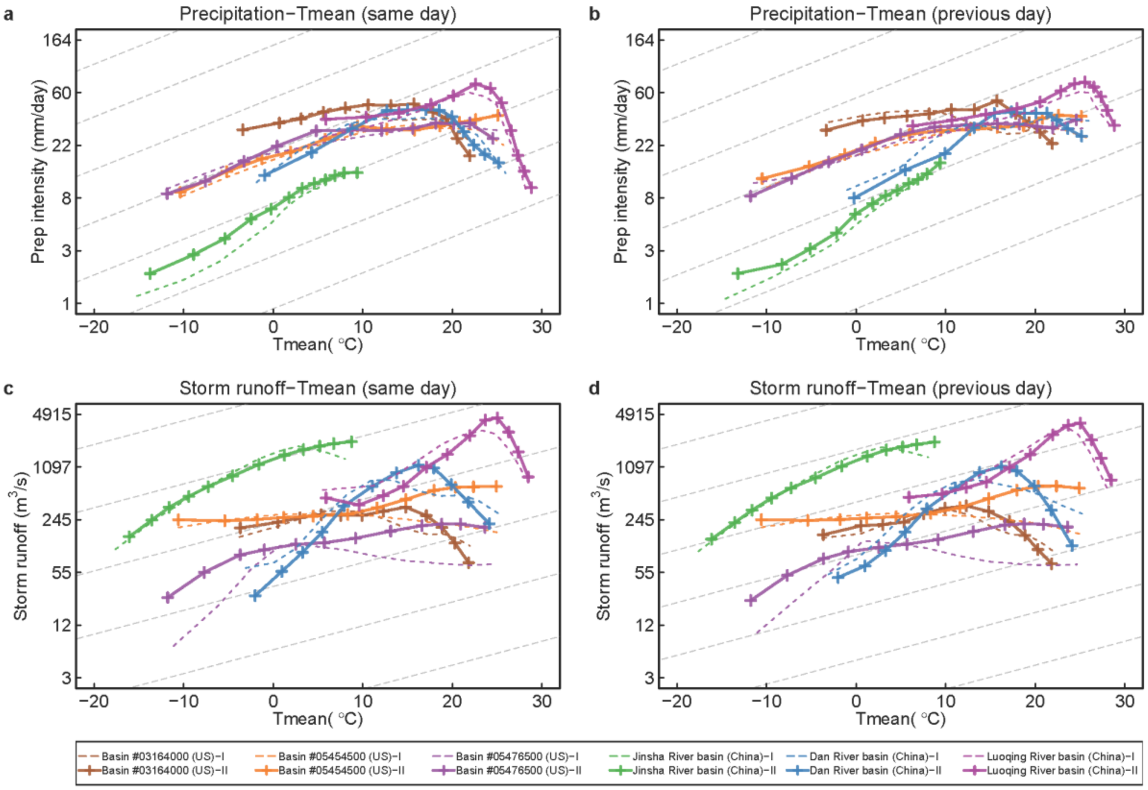

Supplementary Figure 19 | Daily precipitation and storm runoff extremes varying with local temperature during two different periods over six example basins. a-b, scaling curves of precipitation extremes with same-day and previous-day temperature; **c-d**, scaling curves of storm runoff extremes with same-day and previous-day temperature. The example basins are marked in Supplementary Fig. 5a,e. The thin dashed lines are related to Period I (1948-1962 for basins in US, 1961-1975 for basins in China); and the thick solid lines with plus symbols are related to Period II (1989-2003 for basins in US, 2000-2014 for basins in China). The grey dashed lines plot the Clausius-Clapeyron scaling curves as a reference.

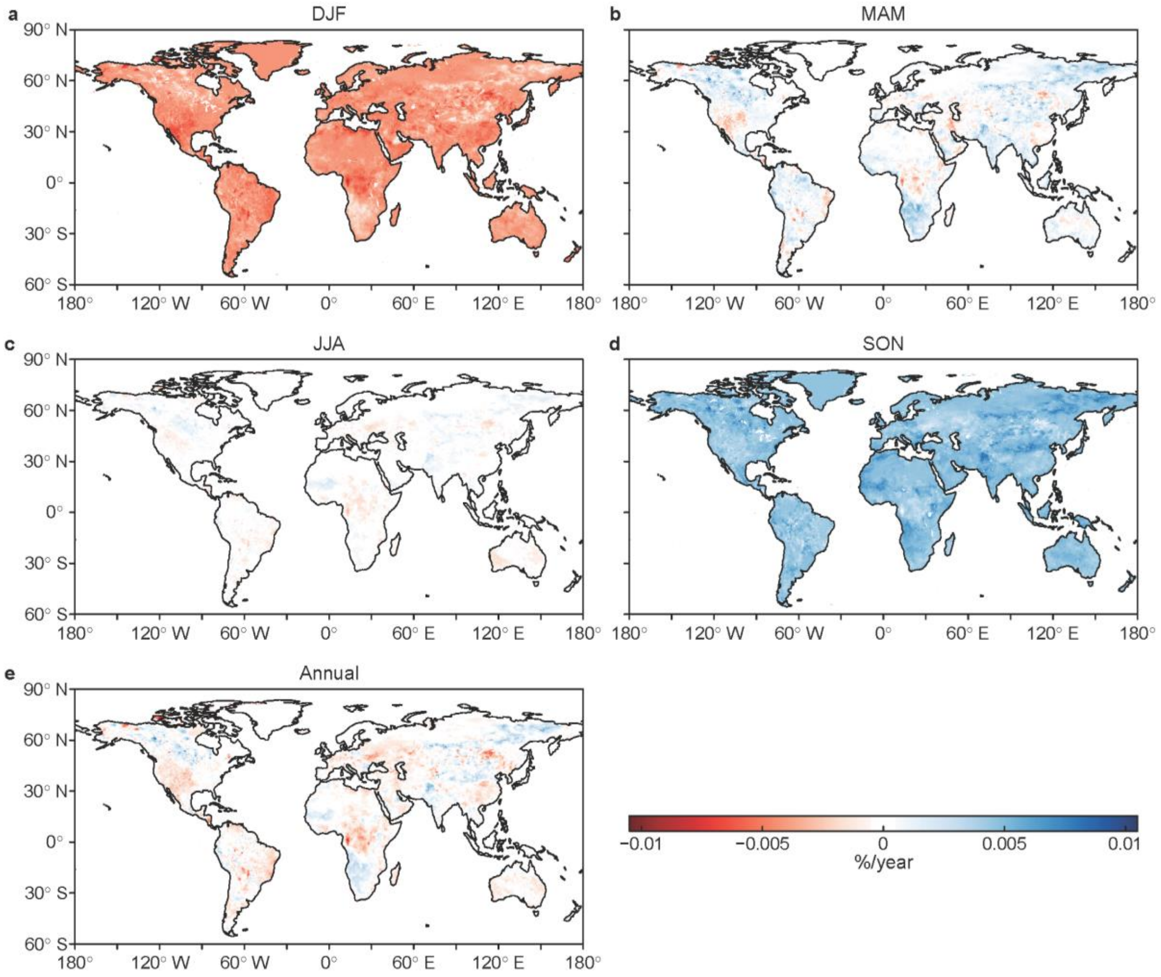

Supplementary Figure 20 | Global trend results of surface soil moisture during 1980-2018. a, trends in winter. **b**, trends in spring. **c**, trends in summer. **d**, trends in autumn. **e**, annual trend results. The soil moisture data is from the Global Land Evaporation Amsterdam Model (GLEAM) version 3.

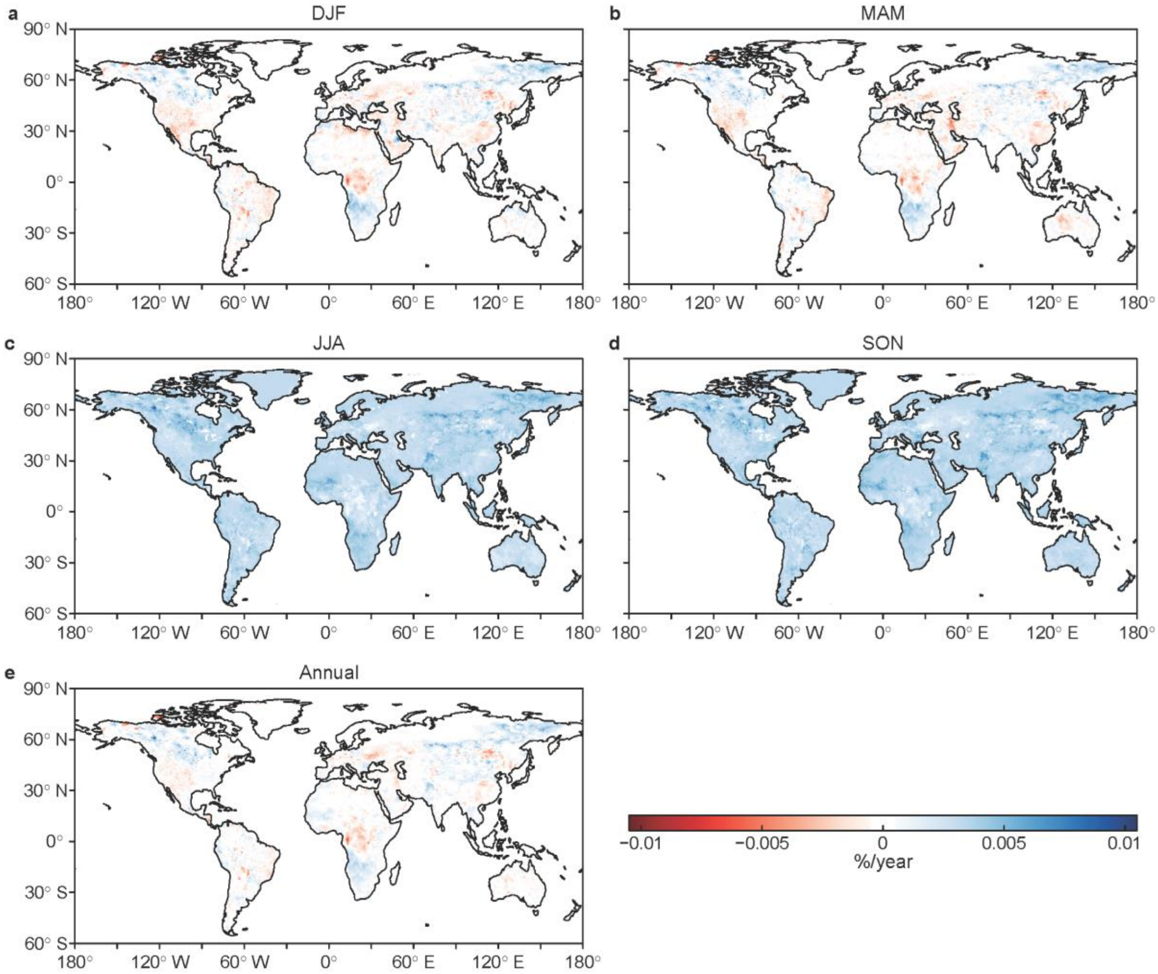

Supplementary Figure 21 | Global trend results of root-zone soil moisture during 1980-2018. a, trends in winter. **b**, trends in spring. **c**, trends in summer. **d**, trends in autumn. **e**, annual trend results. The soil moisture data is from the Global Land Evaporation Amsterdam Model (GLEAM) version 3.

Supplementary Table 1 | Summary of global average scaling rates after omitting data below different snow melt temperatures.

Extreme-Tmean scheme	Snow melt temperature			
	0 °C	1 °C	2 °C	5 °C
99th precipitation -same day Tmean	3.38	3.33	3.30	3.57
95th precipitation -same day Tmean	2.36	2.25	2.17	2.13
99th precipitation -previous day Tmean	4.04	3.98	4.06	4.41
95th precipitation -previous day Tmean	3.43	3.33	3.27	3.24
99th storm runoff – same day Tmean	9.23	8.12	7.52	7.48
95th storm runoff – same day Tmean	9.01	7.92	7.02	6.93
99th storm runoff -previous day Tmean	9.88	8.77	7.99	7.12
95th storm runoff -previous day Tmean	9.60	8.60	7.47	6.96

Supplementary Table 2 | Summary of national average scaling rates under different schemes by two hydrological models.

Extreme-Tmean scheme	US		China	
	GR4J	XAJ	GR4J	XAJ
99th storm runoff – same day Tmean	8.19	8.05	11.34	10.76
99th rain-induced runoff -same day Tmean	7.43	8.00	10.80	10.29
99th storm runoff – previous day Tmean	11.12	9.46	11.61	11.71
99th rain-induced runoff -previous day Tmean	8.32	8.06	11.19	10.68
95th storm runoff – same day Tmean	7.31	7.62	10.53	10.86
95th rain-induced runoff -same day Tmean	7.26	7.53	9.43	10.49
95th storm runoff – previous day Tmean	11.07	9.37	10.91	11.28
95th rain-induced runoff -previous day Tmean	8.12	8.42	9.80	10.53
